# Prevention of Severe Coronavirus Disease 2019 Outcomes by Reducing Low-Grade Inflammation in High-Risk Categories

**DOI:** 10.3389/fimmu.2020.01762

**Published:** 2020-07-14

**Authors:** Radu Tudor Ciornei

**Affiliations:** University Stefan cel Mare of Suceava, Suceava, Romania

**Keywords:** COVID-19, SARS-CoV-2, Coronavirus, probiotics, low-grade inflammation, type 2 diabetes, obesity, cytokine storm

## Introduction

The novel severe acute respiratory syndrome coronavirus 2 (SARS-CoV-2) pandemic infected (as of June 27, 2020) more than 10 million people and claimed 500,000 lives (1). Besides a rapidly rising death toll, this largely unknown disease is accompanied by fear and uncertainty, inflicting worldwide economic consequences, and unpopular lockdown measures. Clinical features of Coronavirus disease 2019 (COVID-19) range from mild respiratory and gastrointestinal symptoms (fever, dry cough, headaches, fatigue, chills, and diarrhea) to severe frank pneumonia and acute respiratory distress syndrome, disseminated intravascular coagulation, and death (2). Besides flu-like symptoms, COVID-19 patients exhibit less common, specific symptoms like loss of smell and taste, chilblain-like lesions on toes, mostly encountered in children (Covid toes), rashes, and skin discolorations (3). Moreover, after initial reports that children develop a low-incidence, asymptomatic infection, with extremely low mortality rate, recent reports described a Kawasaki-like disease with rashes, morbilliform exanthema, pityriasis-like discolorations, fever, reddened tongue, and enlarged coronary arteries (4).

The sudden onset of the COVID-19 pandemic motivated the medical community to decipher the pathophysiological mechanisms underlying severe outcomes rapidly. Developing safe, efficient therapeutic, and preventive strategies against SARS-CoV-2 became a global biomedical priority.

## COVID-19 Pathophysiology

Cellular entry of SARS-CoV-2 is based on high-affinity binding of the spike protein to a cellular receptor named ACE2 (5). Angiotensin-converting enzyme 2 (ACE2) is found on epithelial cells in lungs, arteries, heart, kidney, and intestines. ACE2 is a key player in the Renin Angiotensin System (RAS), which lowers blood pressure by hydrolyzation of vasopressor angiotensin II into vasodilator angiotensin (1–7). A possible physiopathological explanation of the deleterious effects of SARS-CoV-2 is reduced ACE2 function after viral infection, low activation due to massive viral binding and internalization of the receptors, and then cellular pyroptosis. Dysregulation of RAS pathways promotes inflammation and abnormally increases blood pressure and vascular airway permeability (6, 7). Evading cellular defenses, the virus enters a phase of rapid, polymerase-based replication followed by apoptotic cell death, which releases damage-associated molecular patterns like nucleic acids, and ASC protein oligomers (8). In turn, these pyroptotic by-products activate pro-inflammatory alveolar macrophages, triggering the release of a vast array of cytokines and chemokines like Interleukin (IL) 6, IL-7, IL-2, IL-1-β, Tumor necrotic factor (TNF) α, Macrophage Inflammatory Protein (MIP) 1α and MIP-1β, Monocyte Chemoattractant Protein (MCP) 1, and Toll-Like Receptor (TLR) 4 activator protein High Mobility Group Box (HMGB)1 (8, 9). These soluble factors serve as chemoattractants for monocytes, macrophages, and T cells, triggering a self-sustaining, unabated, inflammatory feedback loop. The subsequent hypercytokinemia, followed by vascular epithelium permeabilization, leads to extracellular red blood cell leakage, thromboembolisms, and multiple organ failure. The abnormal elevation of cytokine levels, termed “***cytokine storm***,” sustains the observation that COVID-19 is an immune-based disease. Development of a widely available vaccine, safe for vulnerable categories (immuno-deficient patients, cancer patients, pregnant, and nursing women, etc.) paralleled with immuno-regulatory therapeutic regimens is the two-pronged approach used to harness the pandemic (9–11).

## High-Risk Categories

Early observations revealed that population groups are differentially affected by COVID-19. While the young showed very low prevalence and mortality, the elderly developed severe disease. Also, the death rate is higher among men (2.8%) than in women (1.7%) (12). While age-related effects were hypothetically explained by immunological features, sex-related disparity was speculatively linked to genetic reasons. The *ACE2* gene location on the X chromosome may lead to allelic variants with higher protection of women against COVID-19 (13). Disease progression to Europe showed staggering disparities in the number of deaths per million (Mil) between Western and Eastern countries. While in Italy, France, UK, Spain, Ireland, Belgium, Netherlands, and Sweden, the death rate ranges 350–850/1 Mil (June 27, 2020) in Romania, Slovenia, Serbia, Croatia, Bulgaria, Albania, and Greece the rate is up to 10 fold lower: 22–83/1 Mil (14). The lower death rates (75–150/1 Mil) in Germany, Portugal, and Austria were attributed to early lockdown (Portugal) and to heavy testing in the initial stages and better social distancing compliance and intensive care capacities (Germany and Austria). While a few theories emerged, such as early imposition of quarantine and social distancing measures in Eastern countries, higher life expectancy leading to a greater number of elders and multigenerational living arrangements, discontinuation of BCG vaccine, higher air pollution levels, lower antigenic contact during life, and different social dynamics and population density in Western countries, it is hard to explain a tenfold or higher mortality rate in countries with better-prepared health care systems (15).

A recent study hypothesized that variability in the D/I genotype of the angiotensin-converting 1 (ACE1) enzyme distribution might partly elucidate the variable prevalence of COVID-19 infection amongst continental European countries. The enzyme is characterized by a genetic deletion/insertion (D/I) polymorphism in intron 16 that affects circulating and tissue concentrations of ACE2. High-affinity binding of SARS-CoV-2 to ACE-2 receptors works as a cellular doorway, facilitating viral entry and, subsequently, a higher viral load in the target tissues. According to this study, the D allele, which has higher expression in countries with lower mortality rate, is associated with reduced expression of ACE2, leading to decreased viral entry and less intense disease (16). Also, a high fat, high sugar, and low fiber diet, vitamin D intake, and geographical intestinal microbiota variations were cited as possible protective factors (17, 18). Recent data from the USA and UK underlined a disproportionate effect of COVID-19 on ethnic minorities. The UK's Intensive Care Research Centre reported (April 30) that of 6,574 patients with COVID-19 in intensive care, 1/3 were from non-white backgrounds. In the UK, ethnic minorities account for only 13% of the population. In the US, a similar study published on June 10 confirms the race disparity of COVID-19 victims, with African Americans having a 2, 5-fold higher death risk than Caucasians. Higher incidence of heart disease, diabetes, and asthma, correlated with social circumstances such as lack of medical insurance, poor diet, and higher virus exposure in the line of work, are cited as possible underlying causes of this dire reality.

Besides age, sex, and ethnicity, the presence of comorbidities was a key predictive factor for the outcome of severe COVID-19 cases. A meta-analysis conducted on 1,576 Chinese patients indicated the most prevalent comorbidities of COVID-19 patients: hypertension (21.1%, 95% CI: 13.0–27.2%), diabetes (9.7%, 95% CI: 7.2–12.2%), cardiovascular disease (8.4%, 95% CI: 3.8–13.8%), and respiratory disease (1.5%, 95% CI: 0.9–2.1%). A comparison between severe and non-severe patients resulted in pooled odds ratio (OR) of 2.36 (95% CI: 1.46–3.83), 2.46 (95% CI: 1.76–3.44), and 3.42 (95% CI: 1.88–6.22), respectively, for hypertension, respiratory system disease, and cardiovascular disease (19). Another US study done on 5,350 hospitalized patients between March 1, 2020, and April 4, 2020, in New York City area reported hypertension (56.6 %), obesity (41.7%), and diabetes (33.8%) as main comorbidities. Overall, the median age of patients was 63 years old, and 60.3% were male (20).

## Low-Grade Inflammation

Low-grade inflammation (LGI) is the common feature that encompasses all the high-risk categories for developing severe COVID-19. LGI is characterized by a chronic increase of inflammatory cytokines like IL-6, TNF-α, and IL-1-β. While hyperglycemia, obesity, and hypertension (features of metabolic syndrome, MetS, increasing cardiovascular disease/T2DM risk) are accompanied by LGI, elderly persons develop 3, and inflamm-aging (21–23). Infiltrating pro-inflammatory macrophages in SARS-CoV-2 target tissues (lungs, brain, gut, kidney), and lymphocytes also contribute to the hypercytokinemia that accompanies LGI in MetS patients (22). A recent study revealed that the risk of respiratory failure for patients with IL-6 levels of ≥ 80 pg/ml was 22 times higher than for patients with lower IL-6 levels (24). In high-risk patients with chronic LGI, SARS-CoV-2 infection will elicit a cellular immune response mediated mainly by Th-1, Th-17, and proinflammatory macrophages (10, 11, 25). Even a “normal” pro-inflammatory response may increase cytokine levels further above those of the underlying LGI, hence substantially increasing the risk of developing life-threatening forms of COVID-19. We think that the “**cytokine storm**” term extensively used to refer to COVID-19-induced hypercytokinemia may be better suited for conditions like toxic shock or sepsis. In the case of SARS-Cov-2 infection, a “normal” anti-viral immune response combined with LGI may trigger deleterious effects. However, severe effects of COVID-19 in children and healthy adults without any apparent underlying, LGI-inducing/high-risk conditions still remain unexplained. In addition, scientists are studying the genome of individuals who never became infected with SARS-CoV-2 despite extended exposure. Genome sequencing studies are currently underway in 30 genome sequencing hubs around the world.

## Therapeutic and Preventive Strategies

Beside drug-based approaches, mitigation of disease severity in high-risk categories must include prevention as a first line of defense against COVID-19. Reducing the intensity of LGI in high-risk categories is a realistic goal in diabetics. In 2019, the International Diabetes Federation warned that half of diabetics are still undiagnosed (26). A better effort to diagnose diabetes and hypertension can increase awareness about a higher risk of developing severe COVID-19.

A prospective cohort study, done on 40 newly diagnosed diabetics (aged 45–65) starting prescribed insulin therapy, compared TNF-α and IL-6 levels pre-and post-insulin treatment for 24 and 48 weeks. Volunteers were split into equal cohorts: obese diabetic and non-obese diabetic. Also, 10 age- and sex-matched healthy volunteers were used as a control group. Healthy controls had low levels of TNF-α (4.46 pg/ml) and IL-6 (4.98 pg/ml). Pre-insulin samples of age-matched controls showed an ~20-fold (87.8 pg/ml) increase in TNF-α levels and ~7-fold (34.9 pg/ml) augmentation in IL-6 levels of pre-insulin non-obese diabetics compared to healthy controls (Table 1). The effect of insulin treatment in the same groups was assessed after 24 and 48 weeks of insulin administration. A significant decrease (~1.28 fold in obese and ~1.44 fold in non-obese) was recorded in TNF-α and IL-6 levels after 24 weeks, while after 48 weeks, the downregulation was ~3.63-fold in non-obese and 2.82-fold (Table 1) in obese patients (27). Starting insulin therapy in undiagnosed diabetics can markedly reduce LGI intensity. A retrospective, multi-centered study done in 7,337 COVID-19 cases, among which 952 had pre-existing T2DM, showed that the well-controlled blood glucose (BG) levels group (<10 mmol/L) had a significantly lower in-hospital death rate (1.1 vs. 11.0%) relative to the poorly controlled BG group (28).

**Table 1:** IL-6 and TNF-α levels in obese and non-obese diabetics before and after insulin therapy for 24 and 48 weeks (27).

		**Average IL-6 (pg/ml)**	**Average TNF-α**
Healthy controls		4.98	4.46
	Preinsulin	38.2	112.2
	24 weeks	36.0	105.5
Obese diabetics	48 weeks	20.4	57.2
	Preinsulin	34.9	87.9
Non-obese diabetics	24 weeks	25.5	68.5
	48 weeks	12.3	24.7

The *Firmicutes* to *Bacteroidetes* (F/B) ratio is recognized as a key indicator of dysbiotic states. The F/B ratio changes dramatically in favor of *Bacteroidetes* in obese individuals. Larsen et al. demonstrated that in T2DM patients, *Firmicutes* were significantly higher than *Bacteroidetes*, while in healthy subjects, the number of *Bacteroidetes* remained slightly increased or unchanged. The F/B ratio was significantly and positively correlated with reduced glucose tolerance (29–31). Probiotic and synbiotic supplementation had beneficent effects in T2DM by improving glycated hemoglobin (HbA1c), fasting insulin levels, Homeostasis model of assessment-insulin resistance (HOMA-IR), and the quantitative insulin sensitivity check index (QUICKI) (32, 33). Probiotics play immunomodulatory roles by downregulating LGI levels, skewing pro-inflammatory macrophages toward a pro-restorative phenotype, increasing the Treg/Th17 ratio, and promoting interferon (IFN) γ-producing Th-1 lymphocytes [reviewed in (33, 34)].

A meta-analysis done on 49 articles pooled the known effects of probiotics, resveratrol, Vitamin D, and other molecules on elderly patients with LGI. The study showed that IL-6 levels were downregulated by probiotics (−0.68 pg/ml), Angiotensin Receptor Blockers (−0.37 pg/ml), and omega-3 fatty acids (−0.19 pg/ml) while resveratrol and vitamin D treatments had no significant effects (35).

Besides insulin therapy and probiotic supplementation, other interventional measures like whole-body vibration, increased exercise, and nutritional changes, which aimed to decrease insulin resistance (IR), improve BG control, and restore intestinal eubiosis, were shown to significantly downregulate LGI by decreasing IL-6, TNF-α, and IL-1-β levels (36–40). Although there is no direct evidence certifying a protective effect of supplements like Vitamin C, Vitamin D, Zinc, probiotics, and synbiotics on COVID-19 outcomes, we are hypothesizing that preventive measures targeting LGI can lower the propensity to develop severe disease (41–44). Unlike drug-based therapies, the side effects of supplements are extremely mild (diarrhea, bloating, headaches, antibiotic resistance); however, further studies are necessary to confirm safe usage. These non-drugs based, preventive measures, aiming to downregulate LGI in high-risk patients, must be correlated with risk-minimizing behavior like mask-wearing, social distancing, hand washing, and cough covering. In SARS-CoV2 infection, these supplements may be used only as adjuvant measures recommended by doctors and under no circumstances can replace prescribed drug regimens. A better understanding of the pathophysiology of COVID-19 will enable biomedical scientists to find efficient preventive and therapeutic strategies.

## Author Contributions

The author confirms being the sole contributor of this work and has approved it for publication.

## Conflict of Interest

The author declares that the research was conducted in the absence of any commercial or financial relationships that could be construed as a potential conflict of interest.
